# Development of a Standardised stroke risk assessment for patients with MigraAinous symptoms Reviewed as suspected TIA (SMART): study protocol for a mixed methods study

**DOI:** 10.1136/bmjopen-2025-113253

**Published:** 2025-12-24

**Authors:** Lisa Shaw, Jemma Isaac, Jason Scott, Linda Sharp, Eve Smyth, Lisa Stuart, David J Werring, Christopher Price

**Affiliations:** 1 Stroke Research Group, Population Health Sciences Institute, Newcastle University, Newcastle upon Tyne, UK; 2 Department of Social Work, Education and Community Wellbeing, Northumbria University, Newcastle upon Tyne, UK; 3 Population Health Sciences Institute, Newcastle University, Newcastle upon Tyne, UK; 4 Service User Representative, Contact via: Stroke Research Group, Population Health Sciences Institute, Newcastle University, Newcastle upon Tyne, UK; 5 Department of Translational Neuroscience and Stroke, Uuniversity College London Queen Square Institute of Neurology, London, UK

**Keywords:** STROKE MEDICINE, Observational Study, QUALITATIVE RESEARCH, Migraine, Risk Assessment

## Abstract

**Background:**

Transient ischaemic attack (TIA) and migraine can generate identical symptoms but have very different short-term risks of stroke. Uncertainty about the diagnosis may lead to missed opportunities to prevent stroke if TIA is treated as migraine, or overtreatment if migraine is treated as TIA. This project aims to define the risk of stroke for people with migrainous symptoms reviewed as suspected TIA and develop a risk assessment tool that could promote standardisation of care.

**Methods and analysis:**

The project involves two interlinked studies:

(1) Study A: prospective observational cohort study.

Setting: NHS TIA and stroke services.

Population: adults with migrainous symptoms undergoing review for suspected TIA by a TIA/stroke service and the initial specialist clinician symptom-based diagnosis is either possible migraine or possible TIA with migrainous symptoms.

Data collection: baseline clinical characteristics, investigations and treatments. Stroke, TIA and migraine events within 90 days.

Sample size: 2709 participants.

Main analyses: analysis of stroke risk, development of stroke risk prediction model, preparation of visual tools to represent the risk model.

(2) Study B: qualitative co-design study.

Setting and population: clinicians from NHS TIA and stroke services.

Data collection: focus groups/interviews exploring views about the potential role for a risk assessment tool, the most appropriate visualisation for the risk tool and barriers/facilitators for implementation.

Sample size: approximately 16 clinicians.

Analyses: framework approach using the Implementation Research Logic Model.

**Ethics and dissemination:**

This study has ethical, Health Research Authority and participating NHS Trust approvals. Dissemination of study results will include presentations at national and international conferences and events, publication in peer-reviewed journals, and plain English summaries for patient/public engagement activities.

**Trial registration number:**

ISRCTN16775533.

**STRENGTHS AND LIMITATIONS OF THIS STUDY**

- Study A in this project is a large observational cohort study sufficiently powered to determine the risk of stroke for people with migrainous symptoms reviewed as suspected TIA.
- The inclusion of study B, a qualitative co-design process to inform the visualisation of a risk assessment tool and barriers/facilitators to implementation, will support future adoption into clinical services.
- A limitation of this project is that external validation of the risk prediction model is not planned due to available resources; only internal validation will be undertaken.

## Background

 Stroke affects 110 000 UK citizens each year at a health and social care cost exceeding £9 billion.^1 2^ National healthcare policy aims to reduce stroke-related disability and its wider impact through targeted primary and secondary prevention.^3^ Before a stroke, many patients experience a Transient Ischaemic Attack (TIA), which is a brief episode of neurological impairment due to a temporary reduction in cerebral blood flow. TIA is associated with a 5% risk of stroke after 7 days and 10% during the next 90 days.^4 5^

Although medical treatments cannot completely eradicate stroke risk, meaningful reductions can be achieved.^6 7^ For example, antiplatelet medication produces an absolute risk decrease between 1.1% to 3.6% according to the agent(s) and population.^8^ Therefore, it is important to promptly and accurately identify TIA so that personalised prevention can be initiated and patients given relevant information, such as temporary restrictions on driving. In the NHS, there is a target for patients with suspected TIA to be reviewed by a specialist hospital service within 24 hours of symptoms being reported to initiate appropriate early treatment including antiplatelet, lipid lowering and/or blood pressure control medication, anticoagulation for atrial fibrillation, and surgical endarterectomy if carotid stenosis is present.^6 9 10^

The incidence of first-ever TIA is approximately 50 per 100 000 people per year;^4 5^ however, there are approximately twice this number of referrals to TIA services because of diagnostic uncertainty.^11^ While many TIAs produce classic movement, sensory, speech and visual impairments lasting <20 minutes on a background of vascular risk factors such as hypertension and diabetes, other conditions can cause transient neurological symptoms. Typically half of the referrals made to TIA services are given a different possible or definite diagnosis following specialist review.^11^

Migraine is the single the most common alternative diagnosis among referrals to TIA services. Migraine affects 20% adults of all ages^12^ and is proposed as an explanation for one in eight people referred with suspected TIA.^13^ However, differentiating between migraine and TIA is challenging because of the brief and often subjective nature of symptoms.^13^ Most episodes are not witnessed by a clinician, and by definition, there are no residual physical examination findings. Migraine typically involves a combination of headache, spreading ‘positive aura’ symptoms (eg, visual phenomena including teichopsia, tingling), nausea, increased sensitivity to light and/or sound, and often with a previous history of similar episodes with common triggers (eg, sleep deprivation, hunger, prolonged visual stimulation).^12^ But it is known from longitudinal studies that individuals who regularly experience aura (approximately one third of all migraine sufferers) can also develop ‘negative’ symptoms which are identical to TIA, such as language impairment and limb weakness.^12 14^ During the ‘acephalgic’ variant of migraine, there is little or no headache, whereas headache is reported by up to one third of patients during TIA.^12 15^ Amongst migraine sufferers it has also been shown that genuine TIA can trigger migrainous symptoms,^16^ thereby further increasing diagnostic uncertainty.

Investigations have a limited role in differentiating true migraine from TIA with migrainous symptoms as only 17% of patients with confirmed classic TIA show confirmatory changes of recent cerebral blood flow disturbances on brain imaging,^17^ and following TIA with ‘non-classic’ symptoms estimates vary between 9% and 23%.^17 18^ There is no diagnostic test for migraine. Whilst two diagnostic algorithms for TIA have been developed (the Dawson TIA score^19^ and the Diagnosis of TIA (DOT) score),^20^ neither focuses specifically on migrainous presentations, and neither is used in clinical practice due to concerns about their relevance and accuracy having been developed in single service settings.

Currently, therefore, when patients with migrainous symptoms are being reviewed as suspected TIA, individual clinician judgement based on symptom description and previous medical history is frequently used to determine the diagnosis and management. When there is uncertainty about an important diagnosis, a risk-profile approach may be appropriate to guide treatment, but there is currently no risk-score or algorithm to standardise the interpretation of clinical information for suspected TIA patients with migrainous symptoms. Because migraine is common and can present at any age, it often co-exists with other health problems such as hypertension and diabetes, and therefore a simple demographic stratification approach may lead to both large scale under and over treatment depending on whether the individual clinician places more emphasis on the patient’s symptoms or their vascular risk profile.

Further inconsistency in TIA diagnosis and treatment recommendations may also arise because services are provided by hospital specialists with different backgrounds (eg, neurology, geriatric medicine and acute medicine) whereby speciality-dependent training results in differences in exposure to non-TIA conditions. For example, neurologist training and practice includes diagnosis and management of chronic headache, but this is not the case for geriatric or acute medicine. In addition, TIA services are often supported by nurse specialists and trainee doctors, which may further increase variations in diagnosis and treatment. Service pathways also vary, with some involving initial telephone triage before a standard in-person appointment, whilst full remote assessment by phone and video has been accelerated by the COVID-19 response.^21^

This research will address some of the uncertainty for both clinicians and patients faced with migrainous symptoms reviewed as suspected TIA. As it is currently unclear what information is used in diagnostic decision-making, what treatments are offered, and how stroke risk differs according to the specialist diagnosis assigned, data will be collected to describe current practice and report current stroke risk. The information obtained will then be used to characterise high and low stroke risk groups irrespective of the diagnosis assigned and develop a standardised stroke risk assessment. A standardised stroke risk assessment could be used to guide treatment more effectively than clinical judgement alone.

## Methods and analysis

### Study objectives

1. To describe baseline clinical characteristics, investigations and treatments for people with migrainous symptoms reviewed as suspected TIA, including description according to the specialist clinician view of whether the diagnosis was more likely migraine or TIA with migrainous symptoms.
2. To describe the proportion of people with migrainous symptoms reviewed as suspected TIA who experience at least one clinically documented hospital admission for stroke during the next 90 days and to compare proportions according to the specialist clinician view of whether the diagnosis was more likely migraine or TIA with migrainous symptoms.
3. To report how key features of TIA services influence: the specialist clinician view of more likely diagnosis of migraine or TIA with migrainous symptoms, specific investigations undertaken, treatment recommendations provided, patient outcomes at 90 days.
4. To describe important characteristics of stroke events occurring within 90 days for people with migrainous symptoms reviewed as suspected TIA, including description according to the specialist clinician view of whether the diagnosis was more likely migraine or TIA with migrainous symptoms.
5. To describe important treatment-related side effects occurring within 90 days for people with migrainous symptoms reviewed as suspected TIA, including description according to the specialist clinician view of whether the diagnosis was more likely migraine or TIA with migrainous symptoms.
6. To create a statistical model using clinical characteristics to predict the risk of stroke for people with migrainous symptoms reviewed as suspected TIA irrespective of the specialist clinician view of the more likely diagnosis, and propose at least one matching risk assessment tool.
7. To obtain and incorporate clinician views about the content, format and future implementation strategy of a risk assessment tool.

### Research design

Two parallel studies are being conducted which will interact to synergistically address the study objectives.

Study A: prospective observational cohort study (addresses objectives 1–6).

Study B: qualitative co-design study (addresses objective 7).

### Study A: prospective observational cohort study

#### Setting

The study is taking place within UK NHS hospital TIA and stroke services, selected to reflect a nationally representative range of regions, referral volumes and host specialties (eg, stroke, neurology, care of the elderly, clinical pharmacology).

#### Population

Inclusion criteria:

- Adults with migrainous symptoms reviewed as suspected TIA by a TIA/stroke service.
- Initial review conducted within 1 week of last symptoms.
- Following initial review, a specialist clinician symptom-based diagnosis is either possible migraine or possible TIA with migrainous symptoms.
- At least 40 years old.
- Able to provide informed research consent.

Exclusion criteria:

- Suspected/confirmed TIA without any migrainous features.
- Clear alternative diagnoses after initial review e.g. seizures.
- Previous stroke.
- Referral already made or planned for surgical intervention to prevent stroke e.g. carotid endarterectomy or atrial septal defect closure.
- Currently under secondary care review for the same neurological symptoms.
- Cognitive difficulties preventing consent and/or description of subjective symptoms.
- Life expectancy <90 days.
- Day 90 follow-up is not possible e.g. resident outside of the service boundary.

#### Participant identification and consent

Potential participants are identified by NHS clinicians involved in conducting urgent TIA assessments which cover all of the eligibility criteria listed above. Commonly, TIA services operate as a one-stop hospital-based review, either via attendance at an outpatient clinic appointment or sometimes via an urgent assessment performed by TIA clinicians in the emergency department. Occasionally, TIA reviews are undertaken by telephone/video conferencing and investigations are performed separately.

Once a potential participant is identified as study eligible, a choice of different approach and consent methods are available to the NHS team according to circumstances:

If a potential participant can be approached in person whilst attending a hospital appointment, a research trained member of staff will introduce the study, provide an information sheet and subsequently obtain consent in writing (online supplemental file 1). If a potential participant requires additional time after the appointment to consider study involvement, a study postal consent form and reply-paid envelope will be issued. Telephone follow-up can be undertaken if the postal consent form is not returned, including the option to take consent verbally.

If a potential participant is only undergoing telephone/video-conferencing appointments with the TIA team, the study will be introduced during the consultation and an invitation letter, the information sheet and a postal consent form/reply paid envelope subsequently mailed or emailed according to preference. Telephone follow-up can be undertaken if the postal consent form is not returned, including the option to take consent verbally.

There may also be situations where a rapid review (in person or telephone/video) either prevents the study being introduced or provides very limited time for discussion. In such instances, an invitation letter, the information sheet and a postal consent form/reply paid envelope will subsequently be mailed or emailed. Similar to above, if the postal consent form is not returned, telephone follow-up can be undertaken.

#### Data collection

Study data are collected by NHS staff and entered into an industry standard secure online database managed by the study co-ordinating centre team based at Newcastle University, UK. All data are labelled with a unique study ID number only.

##### Baseline data collection

This study requires a comprehensive description of patient symptoms and to facilitate recall, baseline data collection is performed within 1 week of the TIA review. The data may be collected directly from the patient during the TIA review, from the medical notes made by the clinician at the review, and/or via an additional telephone/video-conferencing/in person discussion held with the participant at the earliest opportunity (and within 1 week of the review).

The information being obtained at baseline is summarised in table 1.

**Table 1:** Data collected at baseline

Clinical setting and assessor	Date of TIA clinical review Setting of TIA clinical review Role and background of specialist clinician who performed the main assessment Specialist clinician view of more likely diagnosis (migraine/TIA with migrainous symptoms) Specialist confidence rating of diagnosis chosen (very high/high/uncertain)
Patient demography	Age Sex Gender Ethnicity Highest educational level
Patient symptoms	Date/time of first onset of symptoms for the study qualifying (referring) event If more than one discrete episode of symptoms has occurred since the study qualifying (referring) event, number of episodes and date/time of last episod For the study qualifying (referring) event and last episode of symptoms before the TIA clinical review, the presence/absence of the following are recorded: Headache including locations affected and pain rating Nausea or vomiting Dizziness, vertigo or pre-syncope Sensitivity to light Sensitivity to noise Vision symptoms, eye affected and type (wavy lines/zig zag lines/flashes/kaleidoscope changes/large blobs/blurry or pixelated vision/seeing items which appeared to be shimmering/seeing items as smaller than normal (micropsia)/seeing items as larger than normal (macropsia)/seeing an image after it has gone (palinopsia)/seeing straight lines as wavy lines (metamorphopsia)/complete loss of vision (blackness)/other) Difficulty using arm(s) including side and severity (weaker or less coordinated than usual/complete weakness) Difficulty using hand(s) including side and severity (weaker or less coordinated than usual/complete weakness) Difficulty using leg(s) including side and severity (weaker or less coordinated than usual/complete weakness) Facial droop (drooping mouth/drooping eyelid) Tingling/pins and needles in face including side Tingling/pins and needles in whole arm(s) including side Tingling/pins and needles in hand(s) including side Tingling/pins and needles in leg(s) including side Numbness/loss of sensation in face including side Numbness/loss of sensation in arm(s) including side Numbness/loss of sensation in leg(s) including side Difficulty talking and type (slurred words/wrong words/no words) Difficulty hearing and type (lost or muffled hearing/ringing or tinnitus/sensitivity to noise) Concentration or memory issues and type (poor concentration/feeling detached/temporary loss of memory) Symptom spread including first/last symptom and speed
Patient medical, social and family history	Medical history (migraine/other headaches/hypertension/high cholesterol/atrial fibrillation/diabetes/angina/myocardial infarction/seizures) Current medication (aspirin/clopidogrel/dipyridamole/ticagrelor/warfarin/dabigatran/rivaroxaban/apixaban/edoxaban/other anticoagulant/statin/other cholesterol treatment/blood pressure medication/anxiety or depression medication) Cigarette/E-cigarette smoker Alcohol consumption Caffeine consumption Family history of headaches or migraine Employment status (working/homemaker/unemployed/in education/retired) Computer/screen use (typically every day/most days/sometimes/rarely/never) Driver For females: pre-menopausal/peri-menopausal/post-menopause/receiving hormone treatment
Specialist initiated investigations undertaken/requested	Blood pressure (systolic value/diastolic value) 12 lead ECG Brain imaging (CT/CTA/MRI/MRA/other) Carotid doppler Transthoracic echocardiogram Portable ECG telemetry (eg, Holter or patch) Other investigation
Specialist initiated treatment	Medication started or recommended (aspirin/clopidogrel/dipyridamole/ticagrelor/warfarin/dabigatran/rivaroxaban/apixaban/edoxaban/other anticoagulant/statin/other cholesterol treatment/blood pressure medication/headache or migraine medication) Endarterectomy referral DVLA advice given Headache service referral Neurology service referral Advice given about managing headaches or migraines Other treatment

CTA, computed tomography angiography; MRA, Magnetic Resonance Angiography; TIA, Transient ischaemic attack.

##### Day 90 data collection

The day 90 data are collected from routine medical records. The information being obtained is summarised in table 2.

**Table 2:** Data collected at day 90

General follow-up from baseline	Results from investigations requested at the study entry TIA clinical review Any diagnosis change between TIA clinical review and day 90 assessment Vital status at day 90 (alive/deceased)
Stroke occurrence	If a stroke(s) has occurred, the following are obtained: Date/time of stroke Stroke symptoms (National Institute of Health Stroke Score) Stroke location (Oxford Community Stroke Project classification) Stroke type (infarction/haemorrhage) Investigations performed with reports (CT/CTA/MRI/MRA/other brain imaging) Treatments received (thrombolysis/thrombectomy/aspirin/clopidogrel/dipyridamole/ticagrelor/warfarin/dabigatran/rivaroxaban/apixaban/edoxaban/other anticoagulant/statin/other cholesterol treatment/blood pressure medication/carotid endarterectomy)
Further TIA and/or migraine symptoms occurrence	If further TIA and/or migraine symptoms have occurred, the following are obtained: Date and time of episode Review by TIA team Review by other service Diagnosis given (TIA/migraine/other) Investigations performed reports (CT/CTA/MRI/MRA/other brain imaging) Treatments received (aspirin/clopidogrel/dipyridamole/ticagrelor/warfarin/dabigatran/rivaroxaban/apixaban/edoxaban/other anticoagulant/statin/other cholesterol treatment/blood pressure medication/treatment for migraine or headaches/carotid endarterectomy) Other neurological events leading to hospital attendance or admission which occurred within the last 90 days
Medication side effects	If new medications were commenced at the study entry TIA clinical review: Free text description of any side effects Medication discontinued

TIA, Transient ischaemic attack.

### Adjudication of stroke diagnosis

In order to ensure that there is confidence in all episodes of stroke, TIA or migraine recorded by participating hospital sites between study entry and day 90, every event will be independently adjudicated by a study panel comprising five stroke or neurology specialists, organised and managed by the study co-ordinating centre team at Newcastle University. The panel will be provided with the clinical features including acute symptoms, clinical radiological reports and treatments received and asked to assign a diagnosis of stroke, TIA or migraine. The panel will not be provided with the diagnosis assigned by NHS site nor the original specialist more likely diagnosis (migraine or TIA with migrainous symptoms) at study entry.

If there is disagreement between the panel and the participating hospital diagnosis, the relevant hospital will be contacted to obtain any additional clinical information, which will subsequently be presented back to the panel. If the disagreement cannot be resolved despite additional information, these cases will be labelled as ‘unclear’ and will not be included in development of the risk assessment.

### Safety evaluation

As this observational study involves only verbal discussion and collection of routinely available data, a safety evaluation is not being conducted.

### Study withdrawal

No specific withdrawal criteria have been pre-set. Participants may withdraw from the study at any time for any reason. Data collected prior to withdrawal will be used in the study analysis.

### Staff training and awareness

Study-specific training is provided for staff at all participating hospitals by the study co-ordinating centre team based at Newcastle University.

### Sample size

The sample size calculation is based on the premise that clinician-assigned more likely diagnosis currently does not separate the study population into groups with significantly different risks of stroke. As it is currently unknown whether the 90 day risk of stroke following a likely diagnosis of TIA with migrainous symptoms is the same as typical TIA (ie, 10%) and how this might be affected by prevention treatments, to power this study the TIA with migrainous symptoms event rate has been conservatively set much lower at 1%. The day 90 risk of stroke in the migraine group has been set at 0.015% based upon the previously reported average incidence of stroke in true migraine sufferers 58/100 000 patients/year.^22^,^24^ At 90% power and a two-sided significance level of p=0.05, a total of 2574 participants (1287 per group) are required to detect this difference (or a greater difference) in stroke rate between specialist assigned more likely diagnosis of migraine and TIA with migrainous symptoms groups. Inflating for a 5% loss in case of routine data access difficulties or allocation of an ‘unclear’ independent adjudication label for any observed outcome events results in an overall target of 2709 participants.

A cohort of this size is also sufficient to examine whether stroke can be predicted by multiple explanatory variables. If the stroke rate in the TIA with migrainous symptoms group is the same as previous TIA cohorts (10%), then at least 128 outcome events are expected.

### Statistical analysis

#### Cohort description and stroke risk

Data will be presented for the total cohort, specialist clinician more likely diagnosis of migraine group and specialist clinician more likely diagnosis of TIA with migrainous symptoms group. This will include:

- Summary descriptive statistics for baseline characteristics, investigations and treatments.
- Proportions suffering first hospitalisation for stroke by 90 days.
- Number and timing of stroke events by 90 days (in case of multiple events).
- Clinical characteristics of stroke events (including clinical and radiological subtype; symptom severity; emergency treatments received).

The proportion of patients experiencing any stroke, defined as at least one clinically documented stroke hospital admission during the next 90 days, will be compared between the specialist clinician more likely diagnosis of migraine group versus the TIA with migrainous symptoms group.

Specialist diagnosis, treatments and 90 day stroke rate across all study patients will also be reported according to:

- The professional background of the clinician performing the assessment.
- The mode of assessment (in person vs remote).
- Other important service characteristics (eg, referral volume).

Exploratory analyses may also consider:

- The clinician confidence rating for their diagnosis (very high/high/uncertain) for migraine or TIA with migrainous symptoms.
- Patients with single versus multiple stroke events.

#### Prognostic model and risk assessment tool development

Development of the risk assessment tool will combine information from both Study A and Study B. Using the stages described by Moons *et al*^25 26^ and applying Transparent Reporting of a Multivariable Prediction Model for Individual Prognosis or Diagnosis (TRIPOD) Statement principles,^27^ a prognostic model will be developed through multiple regression to examine the association between clinical characteristics recorded during initial specialist TIA review (including demographics, symptoms and previous medical history) and the 90 day occurrence of any stroke. The initial model will contain all baseline factors independently associated with day 90 stroke and those nominated as plausible by clinicians during Study B initial co-design rounds. Backward stepwise regression will retain variables for the final prognostic model. The performance of the model will be summarised using calibration and discrimination measures. To correct for overfitting and optimism in model performance, an internal validation using bootstrap samples will be performed which splits the data into training and test samples multiple times.

Approaches to visually represent the prognostic model (eg, nomogram)^28^ will be prepared and shared with clinicians during the final round of the study B co-design process to confirm clinical sense and meaning. These will include simple and more complex risk tool options and their matching predictive performance statistics. Options will include clinician preferences about risk assessment tool format identified during the earlier co-design rounds. The final tool preferred by clinicians is expected to reflect a balance between predictive performance and a format/content which is suitable for real world clinical use^29^ e.g. it can be completed within the time restrictions of clinical care with information that is often available, while retaining acceptable utility.

### Study B: qualitative co-design study

A qualitative co-design study involving two rounds of clinician focus groups and/or individual interviews will be conducted alongside the second half of the prospective cohort study. As well as capturing views from clinicians about the preferred content and format of a risk assessment tool, the Implementation Research Logic Model^30^ will be used as a framework to establish the determinants of implementing the tool into practice, strategies to support implementation, and what indicators could be used to monitor early adoption.

#### Setting

Participating clinicians will be from UK NHS TIA services and focus groups/interviews will be conducted and analysed by qualitative researchers based at Northumbria University.

#### Sample size

Each round will involve up to 16 clinicians. Where possible, the same participants will take part in both rounds but if attrition for round two reaches >20%, additional individuals will be sought.

#### Structure

It is proposed to hold two focus groups (each up to eight clinicians) for each round (ie, 4 focus groups in total). However, if scheduling is problematic, individual semi-structured interviews or smaller focus groups may be undertaken instead. The minimum size for a focus group will be three participants. Both focus groups and interviews will be conducted remotely. Focus groups will be online (eg, MS Teams, Zoom) and interviews either online or by telephone.

#### Eligibility, identification, approach and consent

Purposive sampling will be used to select NHS organisations participating in study A to ensure broad representation of location, service size, characteristics of the patient population, and staff professional and specialty background. Any medical or nursing staff who regularly provide assessment for suspected TIA at the selected organisations will be eligible to participate.

Local principal investigators at the NHS organisations will be asked to identify and approach eligible individuals using a standard email invitation which will ask those interested to contact the qualitative team. The qualitative researchers will supply a participant information sheet, offer to answer any questions and subsequently make the logistical arrangements for individuals willing to take part.

The primary method of consent will be verbal confirmation of agreement, used to minimise time burdens and because focus groups or interviews will be conducted remotely (online/telephone). At the start of any focus group or interview, participants will be reminded about the study details, including the purpose of the focus group/interview and the plan to record, transcribe and analyse data. Thereafter, the recording function will be activated in the technology being used and each participant will be asked to state verbally that they consent to taking part. A verbal consent script will be used to facilitate this process. Although it is expected that most participants will find verbal consent acceptable and convenient, a standard written consent form can be completed if preferred.

If the sample size is not obtained from the initially selected NHS organisations, additional sites may be invited. These may be other sites participating in study A, or, if wider representation is required, new NHS organisations may be approached.

#### Data collection

Topic guides will be used to facilitate discussion, adapted iteratively according to emergent views. All focus groups/interviews will be digitally recorded and transcribed verbatim.

The first round aims to capture experiences and views about current practice for processing patients referred to TIA services with migrainous symptoms, the potential role of a risk assessment tool including the preferred performance characteristics, and which clinical characteristics are used by clinicians to make a judgement about their diagnosis in this scenario. In addition, this round will share examples of risk assessment tools from related clinical areas (eg, cardiovascular events) to stimulate discussion about the content and format for the current clinical scenario and collect views to inform a logic model supporting implementation, including clinical workforce training requirements.

At the start of the second round, findings from round one will be presented back to participants as a validation process and to facilitate discussion about the emerging logic model. The second round will then involve review of the risk tool options that summarise the predictive model from Study A, agreeing a preferred risk assessment format for clinical feasibility, utility and generalisability, and finalisation of a logic model to support future implementation. This approach will increase the probability that, pending satisfactory predictive performance, the tool could be quickly adopted by services.^28 29^ Should the tool not have satisfactory predictive performance for routine use, the findings developed will have sufficient transferability to inform future similar implementation and evaluation work in TIA services.

#### Data analysis

Qualitative data will be analysed using a framework approach^31^ with the Implementation Research Logic Model^30^ providing the structure, while also allowing for inductive themes to be developed. NVivo will facilitate the data analysis process and 20% of data will be independently double-coded to improve rigour.

Analyses will be conducted concurrently throughout study B in a rapid cycle with the qualitative researcher acting as analysis facilitator and participants contributing their views about emergent findings and logic model during each round. Concurrent analysis will mean that on completion of round 2, data collection and analyses will be almost complete subject to final amendments by the research team and final respondent validation. Should there still be any disagreements in the data after round 2, these will be noted and reported.

### Project timeline

Study A commenced participant enrolment in November 2024 and is projected to end in November 2026. Follow-up will subsequently complete in February 2027. The planned start date for study B is November 2026 and end date July 2027.

### Patient and public involvement

This research project involves two public representatives as investigators who were involved in the funding application, study design and contribute to on-going study delivery. In addition, members of the investigator team regularly engage with a local PPI group to discuss study design and progress.

## Ethics and dissemination

Ethical approval for this study was obtained from the London—Stanmore Research Ethics Committee (reference: 24/PR/0977). Health Research Authority and participating NHS Trust approvals are also in place.

Dissemination of study results will include presentations at national and international conferences and events, publication in peer-reviewed journals, and plain English summaries for patient/public engagement activities.

## Discussion

Symptoms caused by migraine and TIA can be identical, and diagnostic tests have a limited role in differentiation. Individual clinician judgement is frequently used to assign the more likely diagnosis and to determine management, and whilst this may involve a stroke risk-profile approach, there is currently no risk-score or algorithm to use for patients with migrainous symptoms being assessed as suspected TIA. As such, there may be both under and over treatment depending on individual practitioner interpretation of clinical information and views about risk. This mixed-methods study aims to define the risk of stroke for patients with migrainous symptoms reviewed as suspected TIA and develop a stroke risk assessment tool that could promote standardisation of care.

Study A has deliberately broad participant inclusion criteria to reflect contemporary clinical practice and a definition of migrainous symptoms or migraine is not imposed to be sure that the study population reflects real world clinician judgement. Participating NHS services will be selected from across the UK to ensure that a representative population is enrolled. Due to the resources available, it is not possible to independently adjudicate radiological investigations to confirm stroke outcome events, however, in order to ensure that events reported by NHS sites are robust, care information provided will undergo independent review. Limited resources also mean that external validation of the risk prediction model is not planned; only internal validation will be undertaken.

To support future adoption into clinical services, the risk assessment tool will be shaped by the views of clinicians who take part in Study B. Although only a small number of professionals will be involved in the co-design, to ensure that the tool has wider transferability, selection for involvement will be from sites with a range of geographical and service characteristics.

At the time of submission of this manuscript in November 2025, 42 NHS sites are involved in study A and a total of 586 participants are enrolled. Study B is yet to commence.

## Supplementary material

10.1136/bmjopen-2025-113253 online supplemental file 1
