# Supplementary material for: Development of a Standardised stroke risk assessment for patients with MigraAinous symptoms Reviewed as suspected TIA (SMART): study protocol for a mixed methods study
Source: BMJ Open. 2025 Dec 24;15(12):e113253. doi: 10.1136/bmjopen-2025-113253 (PMC12742138; doi:10.1136/bmjopen-2025-113253)
Supplement: online supplemental file 1 [file bmjopen-15-12-s001.docx]

**Development of a Standardised stroke risk assessment for patients with MigrAinous symptoms Reviewed as suspected TIA (SMART): study protocol for a mixed methods study**


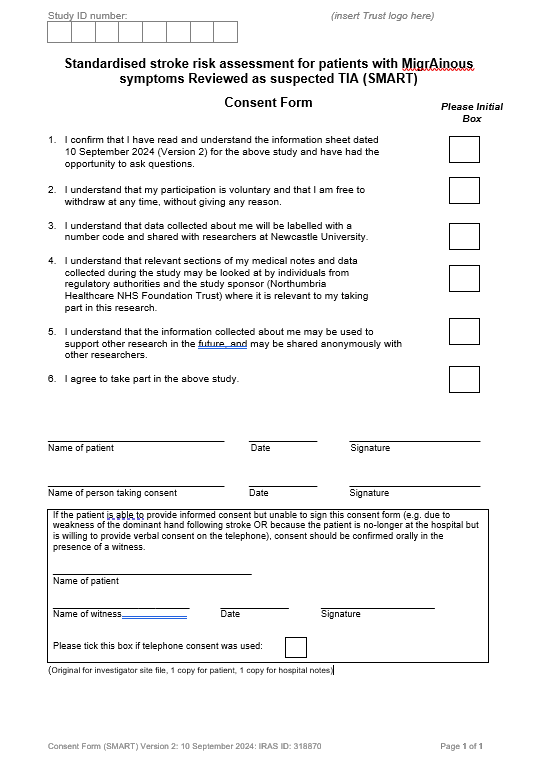
**Supplementary file: SMART study A consent form**
