# Supplementary material for: Development of a Standardised stroke risk assessment for patients with MigraAinous symptoms Reviewed as suspected TIA (SMART): study protocol for a mixed methods study
Source: BMJ Open. 2025 Dec 24;15(12):e113253. doi: 10.1136/bmjopen-2025-113253 (PMC12742138; doi:10.1136/bmjopen-2025-113253)
Supplement: Supplementary file 2 [file bmjopen-2025-113253.reviewer_comments.pdf]

PEER REVIEW HISTORY

BMJ Open publishes all reviews undertaken for accepted manuscripts. Reviewers are asked to complete a checklist review form and are provided with free text boxes to elaborate on their assessment. These free text comments are reproduced below.

ARTICLE DETAILS

Title (Provisional)

Development of a Standardised stroke risk assessment for patients with MigrAinous symptoms Reviewed as suspected TIA (SMART): study protocol for a mixed methods study

Authors

Shaw, Lisa; Isaac, Jemma; Scott, Jason; Sharp, Linda; Smyth, Eve; Stuart, Lisa; Werring, David J; Price, Christopher

---

This article was not externally reviewed at BMJ Open. Protocols that have been independently assessed prior to submission to BMJ Open are usually fast-tracked to publication on the grounds that further substantial changes will not be possible. This independent assessment will usually be external, independent review for both a substantial grant award from a non-commercial or government funder and ethics approval.
